# Inventory and Evolution of Mitochondrion-localized Family A DNA Polymerases in Euglenozoa

**DOI:** 10.3390/pathogens9040257

**Published:** 2020-04-01

**Authors:** Ryo Harada, Yoshihisa Hirakawa, Akinori Yabuki, Yuichiro Kashiyama, Moe Maruyama, Ryo Onuma, Petr Soukal, Shinya Miyagishima, Vladimír Hampl, Goro Tanifuji, Yuji Inagaki

**Affiliations:** 1 Graduate School of Life and Environmental Sciences, University of Tsukuba, Tsukuba 305-8572, Japan; oqo21109797@gmail.com; 2 Faculty of Life and Environmental Sciences, University of Tsukuba, Tsukuba 305-8572, Japan; hirakawa.yoshi.fp@u.tsukuba.ac.jp; 3 Japan Agency for Marine-Earth Science and Technology, Yokosuka 236-0001, Japan; yabukia@jamstec.go.jp; 4 Department of Applied Chemistry and Food Science, Fukui University of Technology, Fukui 910-8505, Japan; yuichirob306@gmail.com; 5 Graduate School of Engineering, Fukui University of Technology, Fukui 910-8505, Japan; harukote.mar@gmail.com; 6 Department of Gene Function and Phenomics, National Institute of Genetics, Mishima 411-8540, Japan; ronuma@nig.ac.jp (R.O.); smiyagis@nig.ac.jp (S.M.); 7 Department of Parasitology, Charles University, Faculty of Science, BIOCEV, 252 42 Vestec, Czech Republic; petr.soukal@natur.cuni.cz (P.S.); vladimir.hampl@natur.cuni.cz (V.H.); 8 Department of Zoology, National Museum of Nature and Science, Tsukuba 305-0005, Japan; gorot@kahaku.go.jp; 9 Center for Computational Sciences, University of Tsukuba, Tsukuba 305-8577, Japan

**Keywords:** DNA replication, family A DNA polymerase, plant and protist organellar DNA polymerase, Trypanosomatida, Kinetoplastea, Diplonemea, Euglenida, Prokinetoplastina

## Abstract

The order Trypanosomatida has been well studied due to its pathogenicity and the unique biology of the mitochondrion. In *Trypanosoma brucei*, four DNA polymerases, namely PolIA, PolIB, PolIC, and PolID, related to bacterial DNA polymerase I (PolI), were shown to be localized in mitochondria experimentally. These mitochondrion-localized DNA polymerases are phylogenetically distinct from other family A DNA polymerases, such as bacterial PolI, DNA polymerase gamma (Polγ) in human and yeasts, “plant and protist organellar DNA polymerase (POP)” in diverse eukaryotes. However, the diversity of mitochondrion-localized DNA polymerases in Euglenozoa other than Trypanosomatida is poorly understood. In this study, we discovered putative mitochondrion-localized DNA polymerases in broad members of three major classes of Euglenozoa—Kinetoplastea, Diplonemea, and Euglenida—to explore the origin and evolution of trypanosomatid PolIA-D. We unveiled distinct inventories of mitochondrion-localized DNA polymerases in the three classes: (1) PolIA is ubiquitous across the three euglenozoan classes, (2) PolIB, C, and D are restricted in kinetoplastids, (3) new types of mitochondrion-localized DNA polymerases were identified in a prokinetoplastid and diplonemids, and (4) evolutionarily distinct types of POP were found in euglenids. We finally propose scenarios to explain the inventories of mitochondrion-localized DNA polymerases in Kinetoplastea, Diplonemea, and Euglenida.

## 1. Introduction

Members of the order Trypanosomatida have been extensively studied because of their pathogenicity to humans. *Trypanosoma brucei*, *Trypanosoma cruzi*, and the species belonging to the genus *Leishmania* cause African trypanosomiasis (sleeping sickness), American trypanosomiasis (Chagas disease), and leishmaniasis, respectively [1]. Besides their significance as the causative agents of deadly diseases, trypanosomatids are important for basic biological research due to the complex architecture of their mitochondrial genomes (mtDNAs) and RNA-editing of mitochondrial transcripts [2]. Trypanosomatids possess a unique mtDNA comprising two types of circular DNA molecule—maxicircles and minicircles—interlocked with one another (so-called kinetoplast DNA or kDNA). A single kDNA contains dozens of maxicircles and thousands of minicircles. Maxicircles carry protein-coding genes and ribosomal RNA genes, of which transcripts need to be edited post-transcriptionally by extensive insertions and deletions of uridines with the help of guide RNAs (gRNAs) transcribed from minicircles. The structural complexity of kDNA is seemingly consistent with a unique set of DNA polymerases required for kDNA replication. In the genus *Trypanosoma*, phylogenetically diverse DNA polymerases were experimentally shown to be localized in mitochondria; (i) PolIA, PolIB, PolIC, and PolID [3] belong to family A, the members of which bear the sequence similarity to bacterial DNA polymerase I (PolI) [4], two of DNA polymerase beta in family X [5], and a DNA polymerase kappa in family Y [6]. Besides PolIA-D in trypanosomatids, several DNA polymerases of family A are known to be localized in mitochondria, such as DNA polymerase gamma (Polγ) in animals and yeasts [7] and plant and protist organellar DNA polymerase (POP), which is also targeted to the plastids of plants and algae [8,9,10].

Trypanosomatida, together with Eubodonida, Parabodonida, Neobodonida, and Prokinetoplastida, are assembled to the class Kinetoplastea [11]. In principal, the characteristics of kDNA (and unique gene expression from kDNA) in trypanosomatids seem to be ubiquitous across the members of Kinetoplastea with modifications [12,13]. In the tree of eukaryotes, Kinetoplastea is further related to the classes Diplonemea and Euglenida, and the family Symbiontida, forming the phylum Euglenozoa [11]. Diplonemid mitochondria contain numerous circular DNA molecules (minicircles) and each chromosome possesses “gene module(s)” that are a piece of the coding regions [14,15]. Both 5′ and 3′ non-coding regions of primary transcripts from gene modules are removed and the resulting transcripts were then assembled into a mature mRNA by *trans*-splicing. After the removal of the 5′ and 3′ non-coding regions described above, transcripts from certain modules undergo substitution RNA editing (cytidine-to-uridine, adenosine-to-inosine and/or guanosine-to-adenosine) and/or appendage RNA editing at the 3′ end (uridine and/or adenosine-appendage) [16]. The architecture of euglenid mtDNA seems to be simpler than those of kinetoplastid/diplonemid mtDNA [17,18,19]. The mtDNA of *Euglena gracilis*, a representative species of Euglenida, is composed of multiple linear DNA molecules, each of which carries one or two full-length genes [18,19]. Although no sequence data are available, the mitochondrion of the euglenid *Petalomonas cantuscygni* was reported to contain multiple DNA molecules in both linear and circular forms based on electron microscopic observation [17]. Finally, our current knowledge of Symbiontida is restricted to morphological information and small subunit ribosomal RNA gene sequences [20,21,22]. Importantly, no systematic survey of mitochondrion-localized DNA polymerases has been done for any of the members of Euglenozoa except trypanosomatids.

In this study, we aim to retrace how the current inventory of mitochondrion-localized DNA polymerases in trypanosomatids has been shaped during the evolution of Euglenozoa. We searched for putative mitochondrion-localized DNA polymerases in diverse euglenozoans. Briefly, we detected PolIA in all of the euglenids, diplonemids, and kinetoplastids examined here, except for a single case of putative secondary loss. PolIB, C, and D are seemingly restricted to members of Kinetoplastea. In addition, we detected novel DNA polymerases, named PolI-Perk1 and PolI-Perk2, and PolI-dipl, all of which are apparently related to but distinct from PolIB-D, in the prokinetoplastid *Perkinsela* sp. and diplonemids, respectively. In euglenids, three distinct types of POP were found and at least two of them were most likely to be localized in mitochondria. According to the inventories of family A DNA polymerases in Euglenida, Diplonemea, and Kinetoplastea, we here discuss the evolution of mitochondrion-localized DNA polymerases in Euglenozoa.

## 2. Results

Pioneering studies demonstrated that the previously described mitochondrion-localized DNA polymerases, namely Polγ, POP, trypanosomatid PolIA–D belong to family A [10,23]. Thus, we surveyed family A DNA polymerases in both public and in-house transcriptome data of four kinetoplastids, four diplonemids, and six euglenids (14 species in total). We repeated the same search against the genome data of two kinetoplatids, *Bodo saltans* and *Perkinsela* sp. As a result, 37 family A DNA polymerases were identified in 14 euglenozoan species and then subjected to phylogenetic analyses along with their homologs, including Polγ, POP, and trypanosomatid PolIA–D. Based on the phylogenetic affinity, we classified the 37 newly identified family A DNA polymerases into nine POP, 13 PolIA, three PolIB, a single PolIC, five PolID, and six “PolIBCD-related” DNA polymerases, named PolI-Perk1, PolI-Perk2, and PolI-dipl (see below).

### 2.1. PolIA is Ubiquitous in Euglenida, Diplonemea, and Kinetoplastea

**Figure 1:**
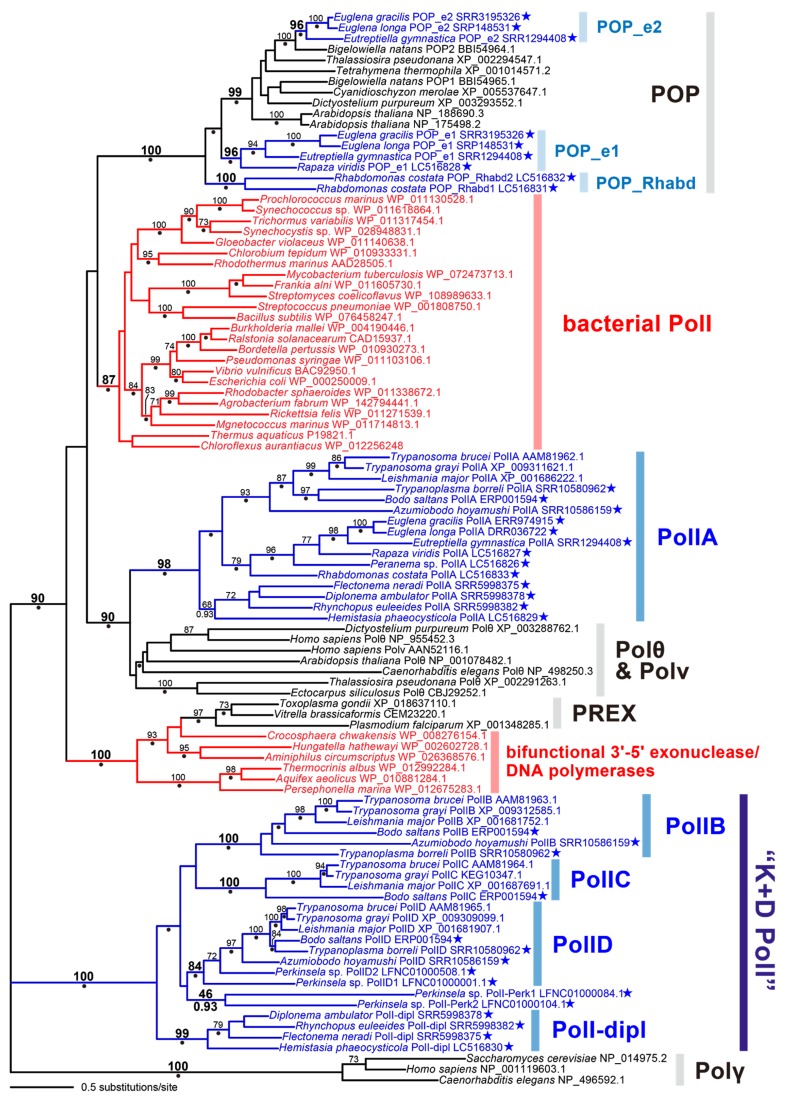
Maximum likelihood (ML) phylogenetic tree of family A DNA polymerases. ML bootstrap values equal to or greater than 70% are shown at the corresponding nodes, except the value for the clade of two *Perkinsela* sequences. Nodes marked by dots were supported by Bayesian posterior probabilities (BPPs) equal to or greater than 0.95, but the BPPs smaller than 0.95 are shown for the nodes of our interest. The bacterial sequences are shown in red. The euglenozoan sequences are in blue. The sequences identified in this study are highlighted by stars.

In the trypanosomatid *Trypanosoma brucei*, four distinct types of family A DNA polymerase—PolIA, B, C, and D—are known to be involved in the maintenance of DNA in their mitochondria [3,24,25,26]. We found that all the species examined in this study (except *Perkinsela* sp.) possess sequences that grouped robustly with trypanosomatid PolIA in the global phylogeny of family A DNA polymerases (Figure 1). We here propose that euglenids, diplonemids, and kinetoplastids (except for *Perkinsela* sp.) possess PolIA, which can be traced back to a single DNA polymerase in the common ancestor of the three classes of Euglenozoa. In the PolIA clade, the homologs of kinetoplastids, diplonemids, and euglenids formed individual subclades, and their monophylies were supported by maximum-likelihood bootstrap values (MLBPs) of 68–93% and Bayesian posterior probabilities (BPPs) of 0.93 to 1.0, while the relationship among the three subclades was not resolved with confidence (Figure 1). The PolIA homologs found in this study were predicted to have the family A DNA polymerase domain (PF00476) at their C-termini (Table S1), as seen in the *Trypanosoma brucei* homolog [3].

**Figure 2:**
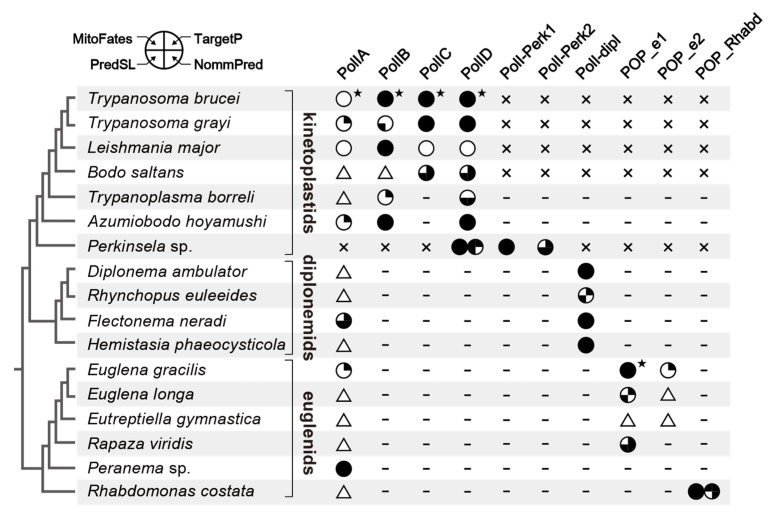
Inventories of family A DNA polymerases in Euglenida, Diplonemea, and Kinetoplastea. For each species examined here, the presence (absence) of each type is displayed by a circle/triangle (a dash/cross). The circles and triangles represent sequences with the complete N-termini and those of which N-termini were absent, respectively. The dashes and crosses represent the absences of homologs in transcriptome and those in both transcriptome and genome, respectively. The sequences with the complete N-termini were subjected to in silico prediction of the mitochondrial targeted signal (MTS) at their N-termini by using TargetP [29], NommPred [30], PredSL [31], and MitoFates [32]. In the case of the MTS being predicted, a subset (or all) of the quarters is filled (upper-right, TargetP; lower-right, NommPred; lower-left, PredSL; upper-left, MitoFates). Stars, which are associated with the four DNA polymerases of *Trypanosoma brucei* and POP_e1 of *Euglena gracilis* indicate the experimentally confirmed mitochondrion-localization. The branching order in the Kinetoplastea clade, that in the Diplonemea clade, and that in the Euglenida clade are based on Yazaki et al. (2017), Tashyreva et al. (2018), and Bicudo and Menezes (2016), respectively [33,34,35].

We recovered the complete N-termini of PolIA homologs in only four kinetoplastids, a single diplonemid and two euglenids out of the 16 homologs examined in this study. None or only one out of the four in silico programs predicted a mitochondrial targeting signal (MTS) at the N-termini of the four kinetoplastid homologs (Figure 2). Although *Trypanosoma brucei* PolIA was shown to be localized in mitochondria experimentally, its N-terminal MTS was not detected by in silico prediction [3]. This likely stems from the difficulty in predicting the mitochondrion-localized proteins in *Trypanosoma brucei* based on the N-terminal amino acid sequences [27]. In contrast, three out of the four programs predicted an MTS in the homolog of the diplonemid *Flectonema neradi*. For the *Euglena gracilis* homolog, only a single program predicted an MTS in its N-terminus. Nevertheless, the study on the *Euglena gracilis* mitochondrial proteome recognized PolIA as a mitochondrial protein [28]. The N-terminus of the *Peranema* homolog was predicted to have an MTS by all of the four programs. The N-termini of the rest of 14 PolIA homologs were incomplete and thus could not be subjected to the MTS prediction (triangles; Figure 2). Considering the robust affinity between *Trypanosoma brucei* PolIA, of which subcellular localization was experimentally confirmed [3], and the other PolIA homologs, we suspect that all of the PolIA homologs identified in this study are mitochondrion-localized proteins.

The precise function of PolIA of *Trypanosoma brucei* has yet to be clarified experimentally [3]. PolIA showed a clear phylogenetic affinity to Polθ that is involved in DNA repair in the nuclear genome (Figure 1) and was postulated to be involved in mtDNA repair [36]. Based on the proposed function of *Trypanosoma brucei* PolIA, this DNA polymerase may be involved in mtDNA repair in diverse euglenozoans.

### 2.2. PolIB, C, D, and “PolIBCD-Related” DNA Polymerases in Diplonemea and Kinetoplastea

Trypanosomatid PolIB, C and D were shown to be closely related to each other but remote from PolIA in previous phylogenetic studies [10,23]. In this section, we describe the distribution and evolution of PolIB, C, D and “PolIBCD-related” DNA polymerases in Euglenozoa. In brief, the sequences which grouped directly with trypanosomatid PolIB, C or D were found only in the kinetoplastids but not in the euglenids or diplonemids examined here.

**Figure 3:**
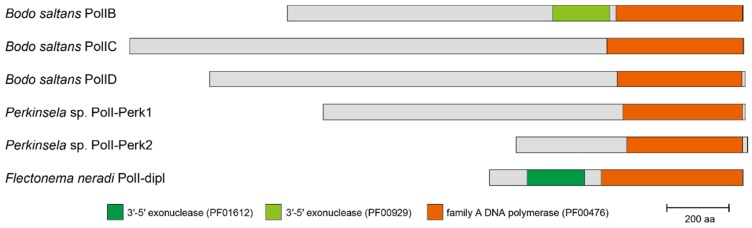
Domain structures of PolIB, C, D, and PolIBCD-related DNA polymerases. The domain structures of PolIB, C, and D are represented by the corresponding homologs of *Bodo saltans* (Note that the N-terminus of *B. saltans* PolIB is incomplete). As PolI-Perk1 and -Perk2 have been undescribed prior to this study, we provide their domain structures. The domain structure of another previously undescribed DNA polymerase identified in diplonemids (PolI-dipl) is represented by the *Flectonema neradi* homolog. Two types of the 3’-5’ exonuclease domains are highlighted in different colors (PF01612 and PF00929 correspond to dark green and light green, respectively). The family A DNA polymerase domains are shown in orange. The detailed domain structures of the kinetoplastid and diplonemid PolIB, C, D, and PolIBCD-related DNA polymerases are described in Table S1.

In Figure 1, trypanosomatid PolID are grouped with the homologs of the eubodonid *Bodo saltans*, the parabodonid *Trypanoplasma borreli*, the neobodonid *Azumiobodo hoyamushi*, and the prokinetoplastid *Perkinsela* sp. together with an MLBP of 84% and a BPP of 0.97, indicating that PolID is ubiquitous in Kinetoplastea. PolIB was detected in all of the orders of Kinetoplastea except for Prokinetoplastida, as *B. saltans*, *Trypanoplasma borreli*, and *A. hoyamushi* appeared to possess DNA polymerases that formed a clade with trypanosomatid PolIB with an MLBP of 100% and a BPP of 1.00. The distribution of PolIC is likely restricted to Trypanosomatida and Eubodonida, as only a single *B. saltans* homolog grouped with trypanosomatid PolIC with an MLBP of 100% and a BPP of 1.00. All of the PolIB homologs appeared to possess both 3′-5′ exonuclease domain (PF00929) and polymerase domain (PF00476) (Figure 3; see Table S1 for the details). Only the polymerase domain (PF00476) was found in the four PolIC homologs assessed here (Table S1). Although *Trypanosoma brucei* PolID has been reported to possess both 3′-5′ exonuclease domain (PF01612) and polymerase domain (PF00476) [3], we detected only the latter domain in the rest of the PolID homologs assessed here (including the homologs of *Trypanosoma grayi* and *L. major*; Figure 3 and Table S1). 

We identified novel family A DNA polymerases in *Perkinsela* sp. and diplonemids, both of which formed a large clade with PolIB, C, and D (Figure 1; labelled as “K+D PolI”). All of the four diplonemids examined here possess the DNA polymerases that formed a clade with an MLBP of 99% and a BPP of 1.00 (designated as “PolI-dipl”), suggesting that this type of DNA polymerase has been inherited vertically from an ancestral diplonemid. Both 3′-5′ exonuclease domain (PF01612) and polymerase domain (PF00476) were conserved in three out of the four PolI-dipl homologs, while the former domain was absent in the *H. phaeocysticola* homolog (only the domain structure of the *Flectonema neradi* homolog is shown in Figure 3; the domain structures of other homologs are provided in Table S1). We found two DNA polymerases in *Perkinsela* sp. (designated as “PolI-Perk1” and “PolI-Perk2”), which were tied together with an MLBP of 46% and a BPP of 0.93 (Figure 1). Only the polymerase domain (PF00476) was found in PolI-Perk1 and PolI-Perk2 (Figure 3 and Table S1). Overall, our phylogenetic analyses failed to resolve the relationship among PolI-Perk1, PolI-Perk2, and four clades of PolIB, C, D, and -dipl with confidence. If we believe the ML tree topology shown in Figure 1, PolI-Perk1 and -Perk2 belong to a novel type of DNA polymerase that is closely related to but clearly distinct from PolIB, C, D, or -dipl. Alternatively, due to the lack of phylogenetic resolution, we cannot exclude the possibility of PolI-Perk1 and -Perk2 (or PolI-Perk2 and -Perk1) being PolIB and C in *Perkinsela* sp., respectively. Unfortunately, we cannot make any definite conclusions on the origins of PolI-Perk1 and -Perk2 in this study. 

We succeeded in recovering the N-termini of all of the PolIC and D homologs examined here, and 10 out of the 12 homologs were predicted in silico to have an MTS by at least two out of the four programs (Figure 2). Among the six PolIB homologs, the complete N-termini were available for all of them except that of *B. saltans*, and MTS was robustly predicted at the N-termini of the *Trypanosoma brucei, L. major* and *A. hoyamushi* homologs. Based on their phylogenetic affinity to the homologous sequences in trypanosomatids (Figure 1) and in silico MTS prediction (Figure 2), we propose that the newly identified PolIB, C, and D are localized in their mitochondria. The N-terminal sequences of PolI-Perk1, -Perk2, and -dipl are available and at least two out of the four programs predicted an MTS in their N-termini (Figure 2). Thus, the novel DNA polymerases found in *Perkinsela* sp. and diplonemids are likely to be localized in their mitochondria.

PolIB, C, and D were experimentally shown to be essential for *Trypanosoma brucei* growth and mtDNA replication in both procyclic and bloodstream forms [3,24,25,26]. Although the difference in function among PolIB, C, and D is poorly understood, their functions in mtDNA replication are unlikely to overlap one another [37]. All we can propose here is the simplest and most conserved scenario—no substantial change in function has occurred to PolIB, C or D through the evolution of Kinetoplastea. Regrettably, the amino acid sequences and domain structures are insufficient to speculate about the precise functions of the novel mitochondrion-localized DNA polymerases in *Perkinsela* sp. and diplonemids, which are absent in trypanosomatids.

### 2.3. POP in Euglenida

**Figure 4:**
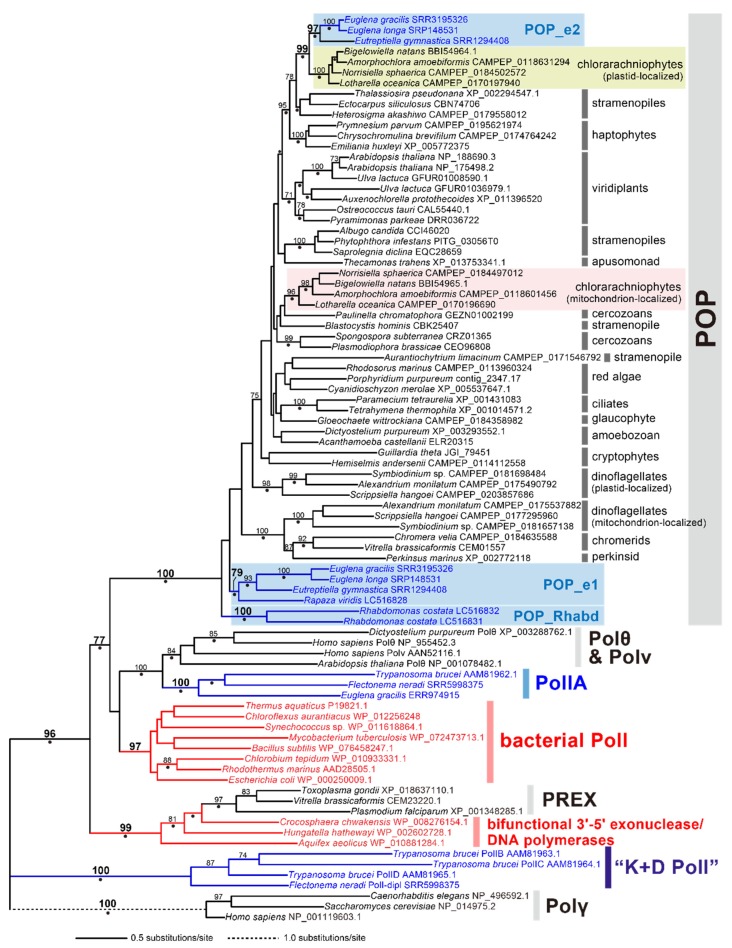
ML phylogenetic tree of plant and protist organellar DNA polymerase (POP). ML bootstrap values equal to or greater than 70% are shown at the corresponding nodes. Nodes marked by dots were supported by Bayesian posterior probabilities equal to or greater than 0.95. The bacterial sequences are shown in red. The euglenozoan sequences are in blue. The three types of POP identified in euglenids (POP_e1, POP_e2, and POP_Rhabd) are shaded in blue. Mitochondrion- and plastid-localized POP in chlorarachniophytes are shaded in orange and light green, respectively.

**Figure 5:**
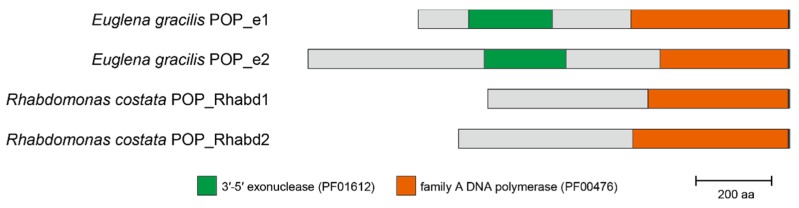
Domain structures of the POP homologs in euglenids. The domain structures of POP_e1 and POP_e2 are represented by the *Euglena gracilis* homologs. The two POP homologs identified in *Rhabdomonas costata* (POP_Rhabd1 and POP_Rhabd2) possess only the family A DNA polymerase domain (shown in orange). The 3’-5’ exonuclease domain (PF01612) is shown in dark green. The detailed domain structures of the POP homologs identified in this study are described in Table S1.

We surveyed family A DNA polymerase sequences in six euglenids, and identified POP homologs from all of the species examined here, except *Peranema* sp. In total, nine POP homologs were found in the five euglenids examined in this study. A phylogenetic analysis of POP alignment separated the euglenid homologs into three distinct types, namely, “POP_e1,” “POP_e2,” and “POP_Rhabd” (Figure 4). *Euglena gracilis*, *Euglena longa*, *Eutreptiella gymnastica*, and *Rapaza viridis* (members of Euglenophyceae) share POP_e1, which grouped together with an MLBP of 79% and a BPP of 0.95. *Rhabdomonas costata* appeared to possess two POP homologs (POP_Rhabd1 and _Rhabd2) that were tied together with an MLBP of 100% and a BPP of 0.99. In the POP phylogeny, the clade of POP_Rhabd1 and _Rhabd2, and that of four POP_e1 homologs were branched subsequently from the root of the entire POP clade (Figure 4). However, because the backbone of the clade is not supported, the sister relationship between POP_e1 and POP_Rhabd cannot be excluded. POP_e2 was identified in *Euglena* spp. and *Eutreptiella gymnastica* and formed a clade with an MLBP of 97% and a BPP of 1.00. The POP_e2 clade was separated from the POP_e1 or POP_Rhabd homologs but grouped with the plastid-localized POP homologs in chlorarachniophytes with an MLBP of 99% and a BPP of 1.00 (Figure 4). As reported for the previously studied POP homologs, POP_e1 and POP_e2 appeared to possess both 3’-5’ exonuclease domain and polymerase domain (Figure 5; see Table S1 for the details). POP_Rhabd1 and _Rhabd2 seemingly lack the 3’-5’ exonuclease domain (Figure 5 and Table S1). 

POP homologs are often localized in both mitochondria and plastids in photosynthetic species [38,39]. The N-termini of three out of the four POP_e1 homologs were completed and predicted to function as an MTS by at least two out of the four in silico programs (Figure 2). On the other hand, neither SignalP [40] nor TMHMM [41] predicted the N-terminal amino acid sequence of *Euglena gracilis* POP_e1 as a typical plastid targeting signal (PTS). Importantly, POP_e1 was detected as a part of the mitochondria proteome [28], while not recognized as the plastid-localized protein [42]. These results consistently suggest that the POP_e1 homologs found in this study are mitochondrion-localized. The two POP_Rhabd homologs were predicted to have an MTS by at least three out of the four programs (Figure 2), suggesting that the two DNA polymerases in *Rhabdomonas costata* are localized in the mitochondria. Based solely on the sequence data, we have little insight into the difference in function between the two mitochondrion-localized DNA polymerases in euglenids, PolIA and POP_e1/POP_Rhabd.

Among the three POP_e2 homologs, we completed the N-terminus of the *Euglena gracilis* homolog. Only a single program predicted an MTS in the N-terminus of the *Euglena gracilis* POP_e2, and this is insufficient to propose its mitochondrial localization. Likewise, no PTS was predicted at the N-terminus of *Euglena gracilis* POP_e2. Indeed, *Euglena gracilis* POP_e2 was recognized as neither a mitochondrial nor plastid protein in the proteomic studies [42]. Thus, we conclude that the POP_e2 homologs are localized in the cytosol.

## 3. Discussion

This study unveiled that PolIA is ubiquitously distributed among Euglenida, Diplonemea, and Kinetoplastea. Thus, we firmly conclude that this type of family A DNA polymerase was obtained in the common ancestor of these three classes and has been inherited vertically to the extant descendants. We also propose that *Perkinsela* sp., which is an obligate intracellular organism of *Paramoeba*, lost PolIA secondarily. There is a room for arguing whether PolIA is absolutely absent in *Perkinsela* sp. However, family A DNA polymerases were surveyed in both the transcriptome and genome data of this species, and there may be little chance to overlook a PolIA gene in the high-quality genome data in particular [43]. Consequently, we propose that a loss of PolIA occurred on the branch leading to *Perkinsela* sp. The conservation of PolIA in Kinetoplastea implies the importance of this DNA polymerase for kDNA maintenance in Euglenozoa. There is a study experimentally demonstrating that PolIA was shown to be dispensable under normal growth conditions in *Trypanosoma brucei* [3]. We suspect that the dispensability of PolIA varies among the life stages of the trypanosome development.

In contrast to the ubiquity of PolIA among Euglenida, Diplonemea, and Kinetoplastea, PolIB, C, and D were identified in the members of Kinetoplastea alone. To our knowledge, no high-quality genome data is publicly available for any of the diplonemids or euglenids. However, it is unlikely that none of the DNA polymerases of interest were overlooked in the transcriptome data of the four diplonemids and six euglenids examined here. Thus, we conclude that PolIB, C, and D are restricted in Kinetoplastea. In addition, we identified PolI-Perk1 and -Perk2 in *Perkinsela* sp., and PolI-dipl in diplonemids, both of which were previously undescribed. As PolIB, C, D, -Perk1, -Perk2, and -dipl formed a “K+D PolI” clade with high statistical support, these DNA polymerases can be traced back to a single ancestral mitochondrion-localized DNA polymerase in the common ancestor of the classes Kinetoplastea and Diplonemea. We here propose that the ancestral DNA polymerase in the two classes was similar to the extant PolI-dipl, and, after the separation of the two classes, the ancestral type of the mitochondrion-localized DNA polymerase has been kept as PolI-dipl in Diplonemea, but has diverged into PolIB, C, D, -Perk1, and -Perk2 during the evolution of Kinetoplastea. So far, it is reasonable to propose that the common ancestor of Kinetoplastea possessed PolID, which was found in all of the members of Kinetoplastea examined in this study. On the other hand, the precise evolutions of PolIB, C, -Perk1, and -Perk2 remain unclear because of two obstacles discussed below.

Firstly, the relationship among PolIB, C, D, -Perk1, and -Perk2 was essentially unresolved in the phylogenetic analyses and makes it difficult to infer how the particular types of DNA polymerase emerged during the divergence of Kinetoplastea. Future phylogenetic analyses with improved sequence sampling may provide a better resolution for the relationship among PolIB, C, D, -Perk1, and -Perk2. Secondly, there is a certain level of uncertainty about the inventories of mitochondrion-localized DNA polymerases in members of Kinetoplastea. The absence of PolIC, -Perk1, and -Perk2 in Neobodonida and Parabodonida needs to be reexamined after the genome data become available from the representative species of the two orders. Likewise, the inventory of mitochondrion-localized DNA polymerases in the class Prokinetoplastida relies entirely on *Perkinsela* sp. in this study. Thus, we need to examine (1) the absence of PolIB and/or C, and (2) the ubiquities of PolI-Perk1 and -Perk2 in members of Prokinetoplastida in the future.

At least one of the three phylogenetically distinct types of POP were detected in all of the euglenids examined in this study, except for *Peranema* sp. It is difficult to conclude that *Peranema* sp. truly lacks any POP homolog, as we only surveyed family A DNA polymerases in its transcriptome data. We predicted that POP_e1 and POP_Rhabd are localized in mitochondria and POP_e2 is a cytosolic protein. Unfortunately, the current data remain uncertain regarding the POP evolution in Euglenida. For instance, we cannot be sure whether the ancestral euglenid possessed a POP homolog for DNA replication in the mitochondrion. If we hypothesize the absence of POP in the ancestral euglenid, a straightforward interpretation of the distribution of the two distinct types of POP over the tree of Euglenida [33] is that POP_Rhabd and POP_e1 emerged separately (1) on the branch leading to *Rhabdomonas costata* and (2) the common ancestor of Euglenophyceae, respectively. In the future, the timing of POP_e1 emergence needs to be revised by incorporating the presence/secondary loss/absence of this type of POP in the early-branching (heterotrophic) species in the tree of Euglenida (e.g., *Peranema* sp.). When the presence/secondary loss of POP_e1 is confirmed in a heterotrophic species, the emergence of POP_e1 should be pushed back to a more ancient branch than that leading to the ancestral euglenophycean species in the tree of Euglenida. In addition, there is a possibility for an alternative scenario assuming that POP_e1 and POP_Rhabd, which were not so distant from each other in the POP phylogeny (Figure 3), evolved from a single POP in the ancestral euglenid. To understand the evolution of mitochondrion-localized POPs in euglenids better, we need to know the inventories of POP in phylogenetically broad euglenids, particularly those of early-branching species.

The proteome data from *Euglena gracilis* suggest that POP_e2 is a cytosolic protein. Although the cytosolic DNA polymerases are not of prime interest in this study, we here discuss the origin of POP_e2 briefly. The POP phylogeny recovered the intimate evolutionary affinity between POP_e2 and the plastid-localized POP in chlorarachniophytes (Figure 3). It is noteworthy that the POP homologs of euglenids and chlorarachniophytes appeared to be distant from those of *Pyramimonas parkeae* and members of Ulvophyceae that are close relatives of the algal endosymbionts which gave rise to the plastids in the two algal groups of interest [44,45]. Thus, no endosymbiotic gene transfer can be invoked in the evolution of POP_e2 in euglenids or plastid-localized POP in chlorarachniophytes. As Euglenida and Chlorarachniophyta are distantly related to each other in the organismal tree of eukaryotes [11], we propose that a POP gene may have been exchanged between the two distant groups, albeit the direction of the gene transfer remains uncertain. Alternatively, an as-yet-unknown eukaryote may have donated a POP gene to Euglenophyceae and Chlorarachniophyta separately. If so, we need to understand the precise diversity of POP in eukaryotes to pinpoint the donor of the ancestral POP_e2 gene.

## 4. Materials and Methods

### 4.1. Sequence Data Preparation

We obtained the transcriptome data of the following members of Euglenozoa from NCBI Sequence Read Archive [46]: Three kinetoplastids (*Bodo saltans*, GenBank accession number ERP001594; *Trypanoplasma borreli* ATCC 50836, SRR10580962; *Azumiobodo hoyamushi*, SRR10586159), three diplonemids (*Diplonema ambulator*, SRR5998378; *Rhynchopus euleeides*, SRR5998382; *Flectonema neradi*, SRR5998375), four euglenids (*Euglena gracilis*, ERR974915, SRR3195326; *Euglena longa*, SRP148531; *Eutreptiella gymnastica* NIES-381, SRR1294408 and *Rhabdomonas costata* PRJNA550357), and a green alga (*Pyramimonas parkeae*, DRR036722). The raw sequence reads were trimmed by fastp v0.19.7 [47] with the -q 20 -u 80 option and then assembled by Trinity v2.8.4 [48]. The assembled genome data of *Perkinsela* sp. CCAP 1560/4 (LFNC01) and *Bodo saltans* strain LakeKonstanz (CYKH01) were downloaded from the GenBank database [46]. We searched for the nucleotide sequences encoding family A DNA polymerases in the 14 assembled transcriptome/genome data described above by TBLASTN [49] using the DNA polymerase domain of *Escherichia coli* DNA polymerase I (KHH06131; the portion corresponding to the 491th–928th amino acid residues) as a query. We retrieved the sequences matched to the query with *E*-values equal to or less than 1 × 10^−4^ as the candidates of family A DNA polymerases.

We repeated the procedures described above on our in-house transcriptome data of the diplonemid *Hemistasia phaeocysticola* and two euglenids (*Rapaza viridis* and *Peranema* sp.). The transcripts encoding the putative family A DNA polymerases were amplified by reverse transcription PCR and the resultant amplicons were sequenced by using the Sanger method. The nucleotide sequences determined in this study were deposited to GenBank/DDBJ/EMBL accession numbers LC516826–LC516833.

### 4.2. Phylogenetic Analysis of family A DNA Polymerases

We found 37 putative family A DNA polymerase sequences in 14 euglenozoan species (four kinetoplastids, four diplonemids, and 6 euglenids) in this study. These sequences were aligned with other family A DNA polymerases, including PolIA, B, C, and D in *Trypanosoma brucei*, *Trypanosoma grayi*, and *Leishmania major*, DNA polymerase γ, θ, and ν, POP, plastid replication and repair enzyme complex (PREX) in Apicomplexa, bacterial PolI, and bifunctional 3’-5’ exonuclease/DNA polymerase [10,23]. These family A DNA polymerases were sampled to include at least three sequences representing each clade in the previous phylogenetic trees. The amino acid sequences were aligned by MAFFT v7.407 [50] with the L-INS-i model. Ambiguously aligned positions were discarded manually, and gap-containing positions were trimmed by using trimAI v1.4 [51] with the -gt 0.9 option. The final alignment comprised 100 sequences with 426 unambiguously aligned amino acid positions. We subjected this alignment to the maximum-likelihood (ML) phylogenetic analysis by IQ-TREE v1.6.12 [52] using the LG + C20 + F + Γ model. The guide tree was obtained by the LG + I + Γ model that was selected by ModelFinder [53]. The statistical support for each bipartition in the ML tree was calculated by 100 non-parametric bootstrap replicates.

The family A alignment was also analyzed with Bayesian method by PhyloBayes v4.1 [54] using the CAT + GTR model. Four Markov chain Monte Carlo (MCMC) chains were run for 25,000 cycles with burn-in of 2500 (maxdiff = 0.144933). Subsequently, the consensus tree with branch lengths and BPPs was calculated from the remaining trees.

### 4.3. Phylogenetic Analysis of POP

We found 9 transcripts encoding POP in 5 euglenid species. Their putative amino acid sequences were added to the alignment that was generated and analyzed in Hirakawa and Watanabe (2019) [10]. In total, 58 POP sequences and 28 family A DNA polymerase sequences belonging to non-POP subfamilies were re-aligned by MAFFT v7.407 with L-INS-i model. After the trimming of gap-containing positions by using trimAI with the -gt 0.8 option, the final “POP” alignment comprised 86 sequences with 509 unambiguously aligned amino acid positions. The ML and ML bootstrap analyses were performed as described above.

The POP alignment was also analyzed with Bayesian method by PhyloBayes v4.1 [54] using the CAT + GTR model. Four MCMC chains were run for 100,000 cycles with burn-in of 10,000 (maxdiff = 0.286478). Subsequently, the consensus tree with branch lengths and BPPs was calculated from the remaining trees.

### 4.4. In silico Prediction of Subcellular Localization and Functional Domains of Family A DNA Polymerases

The mitochondrial localization of the family A DNA polymerases identified in this study were predicted based on their N-terminal sequences by the four different programs, TargetP 1.1 [29], NommPred [30], PredSL [31] and MitoFates [32]. In addition, the *Euglena gracilis* POP_e1 and POP_e2 sequences were subjected to SignalP v3.0 [40] and TMHMM v2.0 [41] to evaluate their plastid localization. Functional domains were searched by HMMER v3.3 [55] with the Pfam database [56].

## 5. Conclusions

In the current study, we provide the inventory of mitochondrion-localized DNA polymerases in phylogenetically broad members of Euglenozoa. The current study demonstrates that the three major classes of Euglenozoa (i.e., Kinetoplastea, Diplonemea, and Euglenida) possess distinctive sets of mitochondrion-localized family A DNA polymerases (summarized in Figure 2). Unfortunately, the inventory of mitochondrion-localized DNA polymerases lends no direct support to solve a ‘big question’ in the evolution of Euglenozoa—how have the distinctive mtDNA architectures emerged and been maintained in Euglenozoa? However, we believe that, in the long run, the results presented here can be a foundation for future studies on the evolution of euglenozoan mitochondria.

To further investigate the early evolution of mitochondrion-localized DNA polymerase inventory and mtDNA architecture in Euglenozoa, the sequence data from the family Symbiontida are indispensable. This lineage was seemingly separated prior to the divergence of Kinetoplastea, Diplonemea, and Euglenida [21,22], but neither the mtDNA data nor genome/transcriptome are currently available. In addition, heterotrophic members of Euglenida are necessary to be studied. This study includes only two heterotrophic euglenids (*Peranema* sp. and *Rhabdomonas constata*), but these species may not be sufficient to represent the diversity of the basal branches in the Euglenida tree [33]. Finally, recent culture-independent studies suggested the presence of previously undescribed lineages that branched after the separation of diplonemids but prior to the divergence of the known kinetoplastids [57,58,59,60,61]. One of these undescribed lineages was found to possess a diplonemid-like mtDNA architecture by sequencing its genome amplified from a single cell (isolate D1) [61]. We retrieved a single family A DNA polymerase sequence, which showed a weak phylogenetic affinity to PolID, in the sequence data of isolate D1 (see the Supplementary Materials). The undescribed lineages mentioned above are critical to understanding the transition of the mitochondrion-localized DNA polymerase inventory and mtDNA architecture in Euglenozoa.

## Supplementary Materials

The following are available online at https://www.mdpi.com/2076-0817/9/4/257/s1, Figure_S1.pdf: ML phylogenetic tree of family A DNA polymerases including the DNA polymerase of the genome data from a single diplonemid-like cell (isolate D1). Table_S1.xlsx: The results of domain searches on the family A DNA polymerases identified in this study. Harada_FamADNApol_ali.nxs: The amino acid sequence alignment in nexus format used for the global phylogeny of family A DNA polymerases (Figure 1). Harada_FamADNApol_ali2.nxs: The amino acid sequence alignment in nexus format used for the global phylogeny of family A DNA polymerases including the sequence of a diplonemid-like organism (isolate D1; Figure S1). Harada_POP_ali.nxs: The amino acid sequence alignment in nexus format used for the POP phylogeny (Figure 3).
