# Supplementary material for: Inventory and Evolution of Mitochondrion-localized Family A DNA Polymerases in Euglenozoa
Source: Pathogens. 2020 Apr 1;9(4):257. doi: 10.3390/pathogens9040257 (PMC7238167; doi:10.3390/pathogens9040257)
Supplement: Supplementary file 1 [file pathogens-09-00257-s001.zip › Harada_supmat2/Figure_S1.pdf]

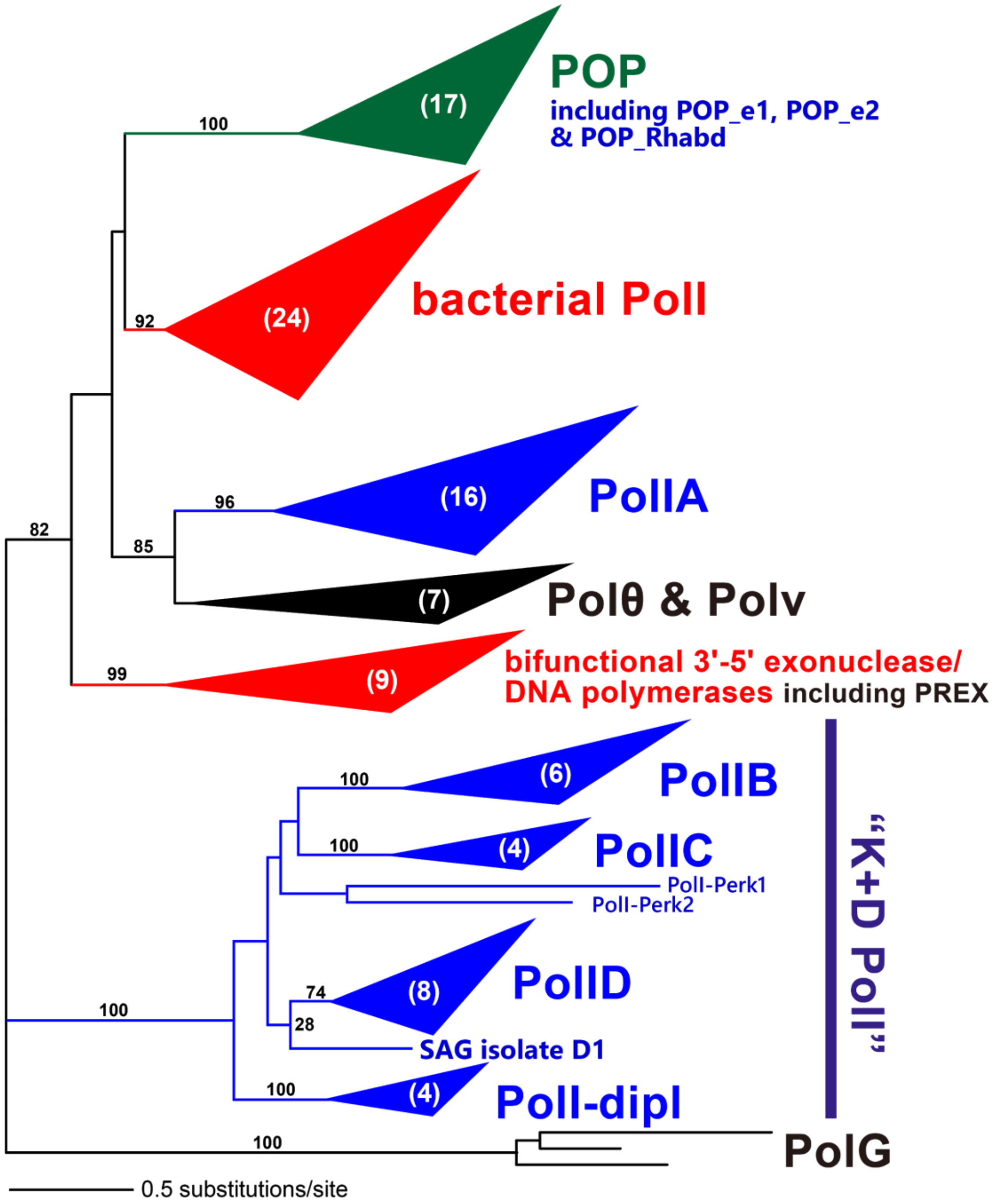

Fig. S1. ML phylogenetic tree of Family A DNA polymerases including D1 PolID. For D1 PolID, canonical introns were manually removed by referencing to the aligned "K+D PolI" sequences. ML bootstrap values equal to or greater than 70% are shown at the corresponding nodes. Clades including four or more sequences are shown as triangles. The number of sequences included in each triangle is in brackets.
